# Editorial: Signaling Proteins for Endosomal and Lysosomal Function

**DOI:** 10.3389/fcell.2021.821719

**Published:** 2021-12-16

**Authors:** Daniel G. S. Capelluto, Cecilia B. Conde, David A. Tumbarello, Geert van den Bogaart

**Affiliations:** ^1^ Protein Signaling Domains Laboratory, Department of Biological Sciences, Fralin Life Sciences Institute, and Center for Soft Matter and Biological Physics, Virginia Tech, Blacksburg, VA, United States; ^2^ Medical Research Institute Mercedes and Martín Ferreyra (INIMEC-CONICET-UNC), Córdoba, Argentina; ^3^ Biological Sciences, University of Southampton, Southampton, United Kingdom; ^4^ Groningen Biomolecular Science and Biotechnology Institute (GBB), University of Groningen, Groningen, Netherlands

**Keywords:** PX domains, TOM1, PLA2, IL6-receptor, FUS, v-ATPase, endosomes, lysosomes

Cells control their activity, in coordination with signal cues, by modulating the levels of plasma membrane receptors and transporters by the action of endosomes and lysosomes. This process is initiated by ubiquitination of cell surface receptors and transporters (cargoes), followed by their internalization by endocytosis. These cargoes are then transported *via* vesicular trafficking to early endosomes and, depending on external cues, they are either recycled back to the plasma membrane or degraded in late endosomal or lysosomal compartments. Given the heterogeneity of endosomes and lysosomes, due to their specific protein and lipid composition, subcellular localization, and membrane shape, it is not surprising they have also arisen as dynamic signaling platforms. Endosomes and lysosomes elicit an array of signaling events, including regulation of gene expression, cell survival, cytoskeletal organization, nutrient uptake, lipid transport, and autophagy (Lefebvre et al., 2018; Norris and Grant, 2020; Yang and Wang, 2021). These organelles can also be hijacked by pathogen effector proteins, facilitating pathogen survival (Asrat et al., 2014).

In this research topic, we assembled review and original research articles that provide recent advances of molecular mechanisms that are a signature for endosomal and lysosomal function under physiological and pathological conditions. We envision that these articles will illustrate a thorough overview of the current progress and future directions relying on proposed mechanistic models.

Peripheral signaling and trafficking proteins target endosomal and lysosomal compartments through interactions with phosphoinositides. One of the best studied phosphoinositide-binding domains is the PX domain, which displays a wide range of specificities for these lipids. Kervin et al. present an elegant meta-analysis that demonstrates that binding of PX domain-containing proteins to membranes is regulated by modification on lysine and arginine residues of PX domains. These side chain modifications, such as acetylation, methylation, malonylation, succinylation, hydroxysobutyrylation, and glycation, neutralize the positive charges on lysine and arginine residues, thus, impairing electrostatic interactions with phosphoinositides. These modifications on PX domains, named MET-stops, are also predicted to interfere with “hot spot” sequences for membrane association as identified by the *Membrane Optimal Docking Area* algorithm. Therefore, MET-stops represent erasers of protein membrane targeting.

Cargo trafficking relies on the function of the cargo sorting machinery known as ESCRT (Migliano and Teis, 2018). Roach et al. provide a detailed review on the function of the endosomal ESCRT protein TOM1 in physiological and disease scenarios. TOM1 participates in cargo trafficking in coordination with other ESCRT proteins and phosphoinositides. TOM1 is also involved in other cellular functions, including autophagy, immune responses, neurodegeneration, and cancer, although many questions regarding molecular interactions in these processes and crosstalk with TOM1’s endosomal function still remain unanswered. Recently, TOM1 has been shown to be hijacked under *Shigella flexneri* infection following accumulation of intracellular phosphatidylinositol 5-phosphate, in which the ESCRT function of TOM1 is impaired. We hope that more research will shed light on the molecular mechanisms governing TOM1 function in these processes.

Schmidt-Arras and Rose-John review the complex spatial regulation of signaling in response to the pro-inflammatory cytokine interleukin 6 (IL-6). The IL-6 receptor consists of the IL-6-binding subunit α and the signaling subunit gp130, which is shared among many different cytokines. The trafficking of the receptors to endosomes and lysosomes is an important layer of regulation. Following endocytosis of the IL-6 receptor-IL-6 complex, signaling can continue from within the lumen of endosomal compartments. Moreover, the receptor can be recycled to the plasma membrane, but also targeted to lysosomes for its degradation. As summarized by Schmidt-Arras and Rose-John, mutations that disrupt endo/lysosomal IL-6 receptor trafficking are a cause for disease.

Dabral and van den Bogaart review the roles of phospholipase 2 (PLA2) in phagocytosis. Phagocytosis is mechanistically related to receptor-mediated endocytosis, and is the process by which immune cells, such as macrophages and neutrophils, ingest pathogens and subsequently kill them in phago-lysosomes. PLA2 is a family of enzymes that hydrolyzes phospholipids, producing a free fatty acid and a lysolipid. In addition to the canonical role of PLA2 in eicosanoid signaling (Soberman and Christmas, 2003), various PLA2 forms play specific roles in endolysosomal function. For instance, the cleavage of lipids by certain PLA2s alters intrinsic membrane curvature, and thereby facilitates the remodeling of the membrane for particle uptake. Similarly, PLA2s play a role in the repairing of membranes to maintain the integrity of endolysosomal compartments.

Trnka et al. present data showing that the protein FUS (Fused in Sarcoma) clusters lysosomes. FUS is an RNA-binding protein involved in regulation of transcription, RNA splicing, and RNA transport (Yamaguchi and Takanashi, 2016). However, mutations in FUS have been related to familial forms of the progressive neurodegenerative disease amyotrophic lateral sclerosis (ALS) (Kwiatkowski et al., 2009). Trnka et al. hypothesized that FUS might affect lysosomal function, since other familial forms of ALS are due to alterations in genes involved in the function of lysosomes and impaired lysosome trafficking has been reported in ALS post-mortem tissue (Root et al., 2021). Indeed, Trnka et al. found that FUS forms cytosolic aggregates in a concentration-dependent fashion and that lysosomes accumulate next to these aggregates.

Acidification within the endosomal pathway is a crucial step to ensure organelle identity, cargo sorting, vesicle fusion, and lysosomal activity. This process is primarily driven by the vacuolar ATPase (V-ATPase), which uses ATP hydrolysis to drive the translocation of hydrogen ions across endosomal and lysosomal membranes resulting in a reduction in lumenal pH. Jaskolka et al. provide a thorough analysis of the yeast RAVE and mammalian Rabconnectin-3 complexes, which have essential roles in the regulation of V-ATPase assembly and organelle acidification. Our understanding of RAVE function and the complex mechanisms associated with V-ATPase assembly in yeast has provided much needed insight into Rabconnectin-3 function in higher eukaryotes, although as highlighted in this review, there are still many open questions. Regardless, it has now become apparent that Rabconnectin-3, *via* its regulation of V-ATPase activity, is an essential player in a variety of intracellular activities including cargo transport and control of signaling along the endocytic route.

In summary, this collection of articles for the Research Topic *Signaling Proteins for Endosomal and Lysosomal Function* provides a more comprehensive understanding of several aspects of the endosomal and lysosomal functions, which will serve as a platform for novel discoveries in these intriguing organelles.
